# An efficacy and mechanism evaluation study of Levosimendan for the Prevention of Acute oRgan Dysfunction in Sepsis (LeoPARDS): protocol for a randomized controlled trial

**DOI:** 10.1186/1745-6215-15-199

**Published:** 2014-06-02

**Authors:** Robert M L’E Orme, Gavin D Perkins, Daniel F McAuley, Kathleen D Liu, Alexina J Mason, Andrea Morelli, Mervyn Singer, Deborah Ashby, Anthony C Gordon

**Affiliations:** 1 Department of Critical Care, Cheltenham General Hospital, Sandford Road, Cheltenham GL53 7AN, UK; 2 Warwick Clinical Trials Unit and Heart of England NHS Foundation Trust, Warwick Medical School, University of Warwick, Gibbet Hill, Coventry CV4 7AL, UK; 3 Centre for Infection and Immunity, Queens University of Belfast, 97 Lisburn Road, Belfast BT9 7BL, UK; 4 Divisions of Nephrology and Critical Care Medicine, Departments of Medicine and Anesthesia, University of California, 521 Parnassus Avenue, Box 0532, San Francisco, CA 94143, USA; 5 School of Public Health, Imperial College London, Norfolk Place, London W2 1PG, UK; 6 Department of Anaesthesiology and Intensive Care, Sapienza University of Rome, Viale del Policlinico 155, 00161 Rome, Italy; 7 Bloomsbury Institute of Intensive Care, University College London, Gower Street, London WC1E 6BT, UK; 8 Section of Anaesthetics, Pain Medicine and Intensive Care, Imperial College London, Charing Cross Hospital, Fulham Palace Road, London W6 8RF, UK

**Keywords:** Levosimendan, Shock, Septic, Multiple Organ Failure, Intensive Care, Critical Care, Randomized Controlled Trial

## Abstract

**Background:**

Organ dysfunction consequent to infection (‘severe sepsis’) is the leading cause of admission to an intensive care unit (ICU). In both animal models and early clinical studies the calcium channel sensitizer levosimendan has been demonstrated to have potentially beneficial effects on organ function. The aims of the Levosimendan for the Prevention of Acute oRgan Dysfunction in Sepsis (LeoPARDS) trial are to identify whether a 24-hour infusion of levosimendan will improve organ dysfunction in adults who have septic shock and to establish the safety profile of levosimendan in this group of patients.

**Methods/Design:**

This is a multicenter, randomized, double-blind, parallel group, placebo-controlled trial. Adults fulfilling the criteria for systemic inflammatory response syndrome due to infection, and requiring vasopressor therapy, will be eligible for inclusion in the trial. Within 24 hours of meeting these inclusion criteria, patients will be randomized in a 1:1 ratio stratified by the ICU to receive either levosimendan (0.05 to 0.2 μg.kg^-1^.min^-1^ or placebo for 24 hours in addition to standard care. The primary outcome measure is the mean Sequential Organ Failure Assessment (SOFA) score while in the ICU. Secondary outcomes include: central venous oxygen saturations and cardiac output; incidence and severity of renal failure using the Acute Kidney Injury Network criteria; duration of renal replacement therapy; serum bilirubin; time to liberation from mechanical ventilation; 28-day, hospital, 3 and 6 month survival; ICU and hospital length-of-stay; and days free from catecholamine therapy. Blood and urine samples will be collected on the day of inclusion, at 24 hours, and on days 4 and 6 post-inclusion for investigation of the mechanisms by which levosimendan might improve organ function. Eighty patients will have additional blood samples taken to measure levels of levosimendan and its active metabolites OR-1896 and OR-1855. A total of 516 patients will be recruited from approximately 25 ICUs in the United Kingdom.

**Discussion:**

This trial will test the efficacy of levosimendan to reduce acute organ dysfunction in adult patients who have septic shock and evaluate its biological mechanisms of action.

**Trial registration:**

Current controlled trials ISRCTN12776039 (19 September 2013)

## Background

Severe sepsis is defined as organ dysfunction consequent to the systemic inflammatory response syndrome triggered by infection. It is responsible for approximately 30% of all admissions to an intensive care unit (ICU) in the United Kingdom (UK) yet, despite improvements in care, the mortality from severe sepsis remains high. According to data from the UK Intensive Care National Audit and Research Centre, the incidence of severe sepsis has increased by 68% over a nine-year period, such that the total number of severe sepsis cases in the UK is in excess of 45,000 per annum with a hospital mortality rate of approximately 45% [1]. Mortality rates increase with an increasing number of organ failures [2,3] whilst the development of acute kidney injury (AKI) doubles the risk of death [4].

### Levosimendan

Levosimendan (Simdax™; Orion Corporation, Espoo, Finland) is a licensed treatment for decompensated heart failure in 48 countries. It sensitizes the myocardium to calcium so that a greater ventricular contraction (and thus stroke volume) can be achieved for the same intracellular calcium concentration. In contrast to catecholamines, levosimendan enhances cardiac performance without significantly increasing myocardial oxygen demand [5]. The drug itself has a short plasma half-life of approximately 1 hour, is approximately 95% bound to plasma proteins and is fully metabolized in the liver and intestine into both active and inactive metabolites. However, the hemodynamic effects are maintained for up to 7 days after a single 24-hour infusion of levosimendan due to the effects of the active metabolite, OR-1896, which has an elimination half-life of approximately 80 hours [6]. Levosimendan also causes vasodilatation, mediated by activation of ATP-sensitive vascular K^+^-channels [7], possesses anti-inflammatory properties [8,9], and has been extensively investigated in patients with acute heart failure due to a variety of etiologies [10].

Myocardial dysfunction is seen in over 50% of patients with severe sepsis [11] and its presence is linked to worse outcomes [12]. Implicated mechanisms include altered calcium trafficking and reduced troponin sensitivity to calcium [13]. The calcium and anti-inflammatory actions of levosimendan provide a strong biological rationale for its use in sepsis. In addition, conventional vasoactive support using catecholamines such as norepinephrine and dobutamine may result in sympathetic nervous system overstimulation and a range of adverse effects [14]. Evidence of a lack of benefit from trials comparing different catecholamine regimens [15], increased mortality in patients exposed to a greater vasopressor load [16], the observation of higher plasma catecholamine levels in non-survivors compared to survivors of critical illness [17], and emerging evidence that beta-blockade may play a role if patients are receiving high-dose catecholamines [18] all provide further evidence of possible harm from conventional catecholamine therapy.

Beneficial effects from the use of levosimendan in severe sepsis have been reported in animal models [19-23]. In humans, several case reports, case series [24-27] and small single-centre clinical trials [28-33] provide good evidence to support the hypothesis that levosimendan may be a promising therapy in severe sepsis. Morelli *et al.* conducted a randomized controlled trial comparing an infusion of levosimendan at 0.2 ug.kg^−1^.min^−1^ to dobutamine at 5 μg.kg^−1^.min^−1^ for 24 hours in 28 patients with septic shock and echocardiographically proven acute left ventricular dysfunction [28]. As well as improving hemodynamics, levosimendan increased creatinine clearance by 64% compared to dobutamine at 5 μg.kg^−1^.min^−1^. In a randomized placebo-controlled trial in 35 patients with septic shock and the acute respiratory distress syndrome, levosimendan at 0.2 μg.kg^−1^.min^−1^ increased cardiac index and reduced mean pulmonary artery pressure compared to placebo [29]. In a further randomized controlled study in 40 patients with septic shock, levosimendan at 0.2 μg.kg^−1^.min^−1^ significantly improved microcirculatory flow when compared to dobutamine at 5 μg.kg^−1^.min^−1^[30]. Two similar studies in septic shock (both randomized controlled trials) demonstrated improved hemodynamics with reduced requirements for additional catecholamines [31] and improvements in cardiac output and mixed venous oxygen saturation [32] with the use of levosimendan. Another trial of 30 patients showed improved hepatic function, measured by indocyanine green clearance, with levosimendan compared to dobutamine at 5 μg.kg^−1^.min^−1^[33]. Further evidence to support an improvement in organ function comes from a retrospective analysis of 99 patients with septic shock who received levosimendan at 0.2 μg.kg^−1^.min^−1^ for 24 hours within 36 hours of admission to the ICU [34]. When compared to matched controls, levosimendan-treated patients demonstrated a 24% increase in glomerular filtration rate at 96 hours together with a reduced peak serum creatinine.

### Risks

Levosimendan has been widely used in patients with acute heart failure with a good safety profile and no known significant pharmacokinetic drug interactions. According to the levosimendan investigators’ brochure, between September 2000 (when the drug first received a license in Sweden) and November 2010 an estimated 440,000 patients have received the drug with a reported serious adverse drug reaction rate of 791 out of 440,000 (0.2%). The most common adverse events reported were hypotension (0.03%) and serious arrhythmias (0.02%). Levosimendan has also been used in over 200 patients with septic shock in published controlled trials and case series without any reported significant adverse effects. Adequate cardiovascular resuscitation with intravenous fluids and norepinephrine, as well as avoiding both the initial bolus loading dose and a high-dose infusion (≥0.4 μg.kg^−1^.min^−1^) help reduce adverse effects when used in sepsis [35]. Levosimendan is currently used in many ICUs within Europe in the treatment of severe sepsis and septic shock and has recently been recommended as an alternative inotrope in the German Sepsis Society guidelines [36].

There is therefore good evidence that levosimendan improves cardiac output, regional perfusion, and other physiological endpoints, including renal function, in patients with septic shock. This trial is therefore designed to identify important clinical outcome benefits and to further explore the mechanisms of action of levosimendan in septic shock.

## Methods/Design

The Levosimendan for the Prevention of Acute oRgan Dysfunction in Sepsis (LeoPARDS) trial is a multicenter prospective, randomized, double-blind, parallel group, placebo-controlled trial. The trial is sponsored by Imperial College London (United Kingdom) and coordinated by the Imperial Clinical Trials Unit. The trial will be conducted in accordance with the principles of the Declaration of Helsinki and Good Clinical Practice and is approved by the independent National Research Ethics Committee London - Harrow (13/LO/0365). It is registered on the European Union Drug Regulating Authorities Clinical Trials database (EudraCT 2012-005159-18) and the International Standardised Randomised Controlled Trial Registry (ISRCTN12776039). The study is funded by the Efficacy and Mechanism Evaluation (EME) program of the National Institute for Health Research (United Kingdom)

The primary objectives of the trial are as follows: (1) to ascertain if levosimendan reduces the incidence and severity of organ dysfunction compared to placebo in adult patients who have septic shock; (2) to identify the effect of levosimendan on individual organ function in septic shock; and (3) to establish the safety profile and pharmacokinetics of levosimendan in this group of patients.

The secondary objectives of the study are: (1) to identify whether levosimendan reduces the need for and duration of catecholamine support and thus reduces myocardial injury; (2) to establish whether levosimendan alters the pro- and anti-inflammatory balance in sepsis; and (3) to collect long-term (3 and 6 month) survival data to help inform the appropriate long-term outcome measure for a subsequent effectiveness trial, should the efficacy of levosimendan be confirmed in this phase IIb trial.

### Inclusion criteria

The target population is adult patients (≥18 years) who require vasopressor support for the management of sepsis despite fluid resuscitation. Patients being treated on an ICU with suspected or confirmed infection will be eligible for the trial if they meet both of the following criteria:

Sepsis - fulfil two of the four criteria for the systemic inflammatory response syndrome (SIRS): (1) fever (>38°C) or hypothermia (<36°C); (2) tachycardia (heart rate >90 beats per minute); (3) tachypnea (respiratory rate >20 breaths per minute or PaCO_2_ < 4.3 kPa) or need for mechanical ventilation; (4) abnormal leukocyte count (>12,000 cells/mm^3^, <4000 cells/mm^3^, or >10% immature band forms).

Shock- hypotension, despite adequate fluid resuscitation, requiring treatment with a vasopressor infusion (such as norepinephrine, epinephrine, or vasopressin analogue) for at least 4 hours and still having an ongoing vasopressor requirement at the time of randomisation.

### Exclusion criteria

The presence of any of the following criteria will prevent entry into the study: more than 24 hours since meeting all of the inclusion criteria; end-stage renal failure at presentation (previously dialysis-dependent); severe chronic hepatic impairment (Child-Pugh class C); a history of Torsades de Pointes; known significant mechanical obstructions affecting ventricular filling or outflow or both; treatment limitation decision in place (such as Do Not Attempt Resuscitation or not for ventilation and/or dialysis); known or estimated weight >135 kg; known to be pregnant; previous treatment with levosimendan within 30 days; known hypersensitivity to levosimendan or any of the excipients; known to have received another investigational medicinal product within 30 days or currently in another interventional trial that might interact with the study drug.

### Primary outcome measure and sample size

This trial is designed to explore the efficacy and mechanism of action of levosimendan, as well as inform a subsequent effectiveness trial if appropriate. We will therefore examine organ dysfunction as measured by the Sequential Organ Failure Assessment (SOFA) score [2] as the primary endpoint (excluding the neurological component). We will compare mean SOFA scores between treatment groups. The mean SOFA in ICUs has been shown to be closely correlated to mortality and its predictive value was similar regardless of length of stay [3].

**Figure 1:**
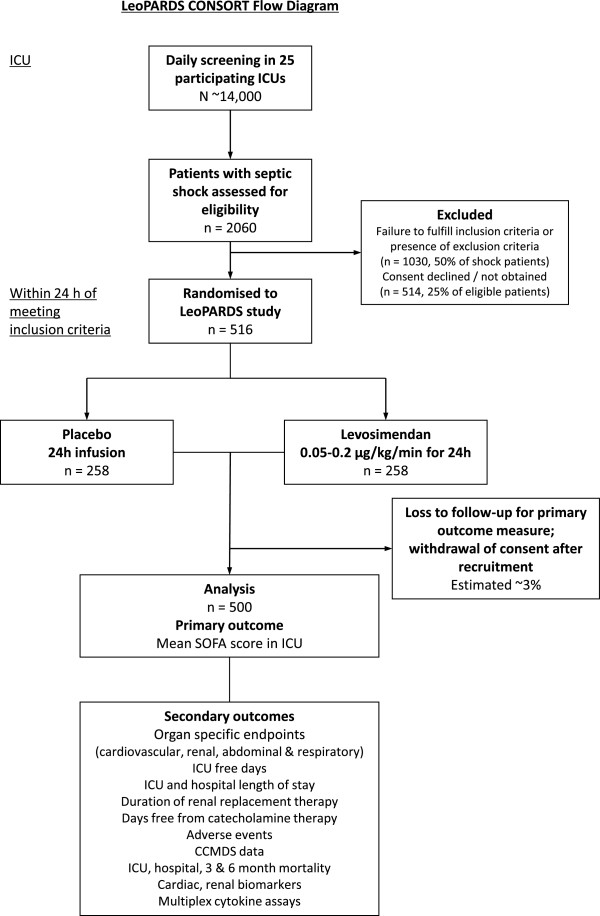
**LeoPARDS proposed trial flow diagram.** CCMDS, Critical Care Minimum Data Set; ICU, Intensive Care Unit; SOFA, Serial Organ Failure Assessment.

Our sample size of 500 patients will provide more than 90% power to detect a 0.5 point difference in the mean SOFA score assuming a SD of 1.5 [3]. In this previous validation study a 1 point rise in the mean SOFA score was associated with a significant mortality increase (mean SOFA 2.1 to 3.0 = 20%, 3.1 to 4.0 = 36.1% and 4.1 to 5.0 = 73.1% mortality; odds ratio 3.06, 95% CI 2.36 to 3.97). We will recruit an additional 3% (16 patients) to account for potential loss to follow-up and withdrawal of consent, as seen in previous UK ICU trials [37], giving a total of 516 patients with 258 in each arm (Figure 1).

### Secondary outcome measures

The SOFA score is a composite of several different organ failures and there may be differential effects of levosimendan in different organ systems. Therefore, in order to gain further insight into the mode of action of levosimendan we will also measure organ specific outcomes.

### Cardiovascular

We will compare oxygen delivery between treatment groups using central venous oxygen saturations (ScvO_2_). This will be measured and recorded at baseline, 6, and 12 hours, and then 12-hourly in all patients with an internal jugular or subclavian central venous catheter (CVC) for up to 96 hours, if the CVC remains *in situ*. The study will have more than 95% power to detect a 5% difference in ScvO_2_ assuming a SD of 15%.

Cardiac output monitoring using specified devices (PiCCO™, LiDCOplus™, esophageal Doppler, or pulmonary artery catheter) will be measured in select centers, at baseline, 6, 12, and 24 hours, and then 12-hourly for up to 96 hours or as long as the device is clinically required if shorter. Based on cardiac output data from the Vasopressin and Septic Shock Trial (VASST), a subgroup of 150 patients will provide more than 85% power to detect a 0.66 l.min^−1^.m^−2^ difference in cardiac index between treatment groups over this period assuming an SD of 1.3 [38]. We will also examine the effect of levosimendan in the subgroup of patients who have a low cardiac output at baseline, as evidenced by a low ScvO_2_ or measured cardiac output.

### Respiratory

Recent evidence has suggested levosimendan may also enhance calcium sensitivity in the diaphragm muscle and thus improve diaphragm contractility [39]. In those patients who require intubation and mechanical ventilation we will assess time to successful liberation from mechanical ventilation. This will be defined as: (1) extubated with face mask, nasal cannulae or room air, or (2) T-piece/heat and moisture exchange (HME) filter breathing, or (3) tracheostomy mask breathing, or (4) CPAP breathing ≤5 cm H_2_O without any pressure support or mandatory ventilation.

A total of 450 patients (90% of the sample size of 500) will provide 80% power to detect a hazard ratio of 1.4, based on the assumption that 63% of patients will be successfully liberated from mechanical ventilation by day 28.

### Renal

In view of the data demonstrating a beneficial effect of levosimendan on renal function in sepsis we will compare stages (0, 1, 2, and 3) of AKI using the Acute Kidney Injury Network (AKIN) definition [40]. Since death is clearly a worse outcome than renal failure, patients who have died will be categorized into a fifth stage. These five groups at day 14 will be analyzed as ordinal categorical data, using a constrained non-proportional odds model such that the log-odds ratios are assumed constant across all the cumulative probabilities except the last (death).

Power calculations have been undertaken using simulation with multi-state modeling based on previous data from previous septic shock trials [41-43]. These simulations suggested that the maximum difference in rates of AKI and death between treatment groups might be seen around day 14. The trial will have between 65 and 90% power to detect between 25 and 35% improvement in renal function with levosimendan.

### Secondary mechanistic outcomes

Serial blood and urine samples will be collected from patients at baseline, day 2, 4 and 6. Assays will include markers of AKI, myocardial dysfunction, inflammation, and intestinal perfusion. Possible assays are given below but as biomarker discovery is a very active area of research we will re-evaluate the best assays available at the time that study enrolment is complete and consider the use of additional or complementary biomarkers. Current possible assays include (1) AKI biomarkers in urine and plasma. Urinary Neutrophil Gelatinase-Associated Lipocalin (NGAL) is currently the best validated biomarker of AKI [44]. Alternative biomarkers of AKI (such as Kidney Injury Molecule-1 or cell cycle arrest biomarkers [45]) may be better characterised and validated by the end of the trial. (2) Markers of myocardial dysfunction. Serum B-type Natriuretic Peptide (BNP) has been demonstrated to be a reliable biomarker of myocardial injury, ischemia, and dysfunction in septic patients and also as a prognostic marker for a poor outcome [12,46]. (3) Biomarkers of systemic inflammation. This will be measured using a multiplex inflammatory biomarker assay. This will allow an assessment of the pro- and anti-inflammatory cytokine balance over time in patients with septic shock and allow a more detailed study of the other potential mechanisms of action of levosimendan. In addition, samples will be stored for subsequent analysis as appropriate.

### Pharmacokinetic substudy

The initial 80 patients enrolled in the study will have an additional 3 ml of blood collected while on ICU on days 2, 4, 6, 8, 10, 13 and 16 for assays of levosimendan and its active metabolite OR-1896. We will then compare the area under the curve (AUC) and the maximum concentration (C_max_) between patients with and without AKI and also with previous pharmacokinetic data from other studies. The drug and metabolite levels will be correlated to possible adverse hemodynamic events, namely tachycardia and hypotension. The independent Data Monitoring and Ethical Committee (DMEC) will be asked to suggest alternative dosing regimens if drug or metabolite levels are unexpectedly high in certain patient groups or if there is an association between infusion rates, drug levels, and adverse events. They will advise if any alternative dosing regimens should alter the sample size for the trial.

## Trial conduct

### Consent

All patients in ICU who are clinically judged to have severe sepsis will be screened against the inclusion and exclusion criteria. Informed consent will be obtained prior to enrolling patients into the trial. In most cases it will not be possible to obtain prospective consent from the patient due to a lack of capacity as a result of their critical illness and its treatment. Under such conditions, written informed consent will be sought from the patient’s personal legal representative (PerLR) who will be provided with a copy of the Patient Information Sheet (PIS). If the patient lacks capacity and there is no PerLR then a doctor, unconnected with the trial, may act as a professional legal representative (ProLR) and provide consent for enrolment into the trial. All surviving patients will be informed about the trial once they regain capacity and will be asked to give consent to their ongoing involvement in the trial.

### Randomization and supply of study drug

Orion Corporation (Espoo, Finland) is providing the study drugs for the trial. Levosimendan 12.5 mg/5 mls vials and identical placebo will be supplied to Victoria Pharmaceuticals (Belfast, UK) who will be responsible for packaging, labelling, and distributing the drugs to study sites in accordance with Medicines and Healthcare products Regulatory Agency (MHRA) requirements.

Randomization will be by computer generated randomized numbers and will use an online system (InForm™, Oracle Corp, California, United States). Randomization will be stratified by ICU and will occur on a 1:1 basis in permuted blocks.

### Administration of study drug

**Figure 2:**
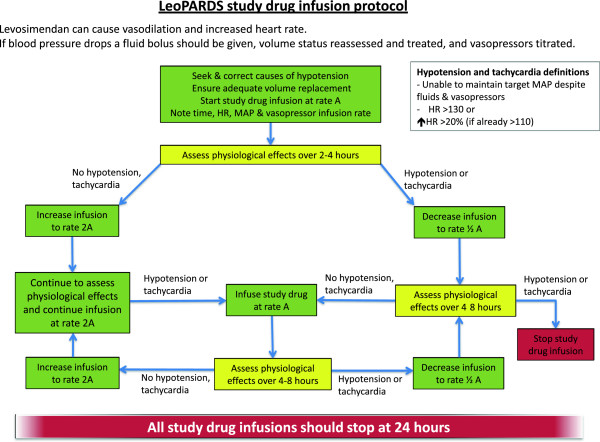
**LeoPARDS blinded study drug infusion management guideline.** HR, Heart rate; MAP, Mean Arterial Pressure. ⬆ = increased.

Patients will be randomized to receive an intravenous infusion of levosimendan at 0.05 to 0.20 μg.kg^−1^.min^−1^ or placebo for a period of 24 hours. The infusion will be prepared by diluting the contents of one vial of study drug in 500 ml of 5% dextrose. The study drug will not be started until the treating clinician is confident that adequate fluid resuscitation has been achieved and that the patient has reached their target blood pressure; this may be a mean arterial pressure (MAP) of 65 to 70 mmHg but may be increased or reduced if the treating clinician feels this is clinically necessary. The infusion will then be commenced at a rate of 0.1 μg.kg^−1^.min^−1^ based on actual (estimated) body weight. Although the summary of product characteristics states that an initial bolus of levosimendan should be given, in this trial the bolus dose will not be given to avoid hypotension and maintain patient safety. After 2 to 4 hours the infusion will be increased to 0.2 μg.kg^−1^.min^−1^ for the remaining 20 to 22 hours if there is no significant hypotension or tachycardia. Any initial hypotension will be treated with a fluid challenge and an increase in vasopressor dose if needed. Throughout the duration of the infusion, clinicians will have the option to reduce the infusion or stop it if they deem there to be significant hypotension or tachycardia. Details of the study drug infusion protocol are shown in Figure 2. Titration of the study drug dose between 0.05 and 0.2 μg.kg^−1^.min^−1^ will ensure patients receive an effective dose whilst minimizing adverse effects.

### General patient management and treatment

Patients will receive all normal standard care and be managed in accordance with current accepted best practice as recommended by the Surviving Sepsis Campaign (SSC) guidelines [47]. Crystalloid solutions will be used for intravenous fluid resuscitation. Gelatin-based or human albumin solutions may be used as alternative resuscitation solutions. Norepinephrine is the initial vasopressor of choice. This should be titrated to a target MAP of 65 to 70 mmHg or as deemed appropriate by the treating clinician. Vasopressin, or any of its analogues, may be used as an alternative vasopressor or in addition to norepinephrine. The lowest dose of vasopressor that maintains a MAP that provides acceptable tissue perfusion should be used. Additional inotropic drugs may be used as clinically indicated. Dobutamine is the inotropic agent of choice but others, such as epinephrine or milrinone, may be used. A central venous saturation (S_cv_O_2_) of more than or equal to 70% should be targeted in the early stages of septic shock management. Dobutamine (or any other inotropes) should be titrated down and weaned off once an adequate oxygen delivery is achieved. Hydrocortisone should only be used for patients who are poorly responsive to vasopressors, as per the SSC guidelines [47]. Low doses, such as 200 mg hydrocortisone administered intravenously daily in divided doses or by infusion, should be used and weaned off once shock resolves.

A lung-protective strategy should be used: target tidal volume 6-ml.kg^−1^ ideal body weight, limiting plateau pressure to ≤30 cmH_2_O, accepting permissive hypercarbia, and ensuring adequate levels of positive end-expiratory pressure (PEEP). High frequency oscillation, neuromuscular blockade, inhaled nitric oxide, prone positioning, and extra-corporeal membrane oxygenation are all permitted as needed for the management of refractory hypoxemia. Continuous veno-venous hemo(dia)filtration is the renal replacement therapy of choice, however, other modalities such as intermittent hemodialysis may be used since there is no clear evidence that one modality is superior [48]. High volume hemofiltration solely for the treatment of sepsis in the absence of renal failure should not be used.

### Pharmacovigilance & patient follow-up

An assessment of the safety of levosimendan is an important outcome measure for the LeoPARDS trial. Given that the trial is being conducted in critically ill patients, a significant number of patients are likely to experience adverse events (AEs). Events that are in keeping with the patient’s clinical condition will not be reported as AEs. The following clinical outcomes from sepsis will not be recorded as AEs unless the local investigator deems the event to be related to the study drug or the protocol: death, cardiovascular failure, respiratory failure, hepatic failure, renal failure, and hematological failure. Specific information about the occurrence of a myocardial infarction or acute coronary syndrome and life-threatening arrhythmias will be recorded in the case report form. All adverse events will be assessed for likely relationship to the study drug. All serious adverse event (SAE) or a suspected unexpected serious adverse reaction (SUSAR) will be reported to the trial coordination centre within 24 hours and to the relevant authorities in accordance with current regulations.

Patients will be followed up on daily whilst on the ICU. Routinely collected clinical data (cardiovascular, respiratory, and renal physiological variables as well as hematological and biochemical blood test results) will be recorded on a daily basis during this time using the electronic case report form. Patients will also be followed up to ascertain survival status at 28 days post-recruitment, at hospital discharge, and at 3 and 6 months post-recruitment using The Health and Social Care Information Centre.

### Statistical analysis and trial oversight

A full statistical analysis plan will be drawn up before the trial database is locked and analyzed. Prior to performing any statistical tests or fitting statistical models, an exploratory analysis of the baseline variables and outcome measures will be completed. The level and pattern of missing data in the baseline variables and outcomes will be established by forming appropriate tables and likely causes of any missing values will be investigated. Any issues will be dealt with using standard statistical techniques. The primary analyses will be carried out on an intention-to-treat basis. The Trial Steering Committee (TSC) with an independent chair and a majority of independent members including patient and public representatives will be responsible for overseeing the progress of the trial and will meet six-monthly. The independent DMEC will meet six-monthly to review ongoing recruitment, protocol compliance, clinical outcome, and adverse event data. They will provide the TSC with written reports advising about ongoing trial conduct.

The trial will end once 516 patients have been enrolled, all patients have completed 6 months follow-up and the database is locked.

## Discussion

There is good evidence from pre-clinical studies [19-23], case series [24-27], and small clinical trials [28-33] looking at physiological endpoints that levosimendan may have a beneficial role in the management of septic shock. Its mechanism of action as a calcium sensitizer means it has theoretical advantages over other inotropic drugs, including less increase in myocardial oxygen demand and reduced rates of arrhythmias. It may also improve peripheral organ perfusion and have anti-inflammatory properties.

This trial will test the clinical efficacy of levosimendan to reduce acute organ dysfunction in adult septic shock and will also examine its safety profile in this patient group. The primary outcome measure (mean SOFA score) allows an overall assessment of the effect of the drug on organ function which is known to correlate with survival. However, as a composite score it is vital to examine each organ system separately to fully understand the biological effect of levosimendan. The collection and analysis of blood and urine samples will also allow a fuller examination of its biological effects.

## Trial status

The trial is due to open for recruitment in January 2014. Recruitment of all 516 patients is expected to take 30 months.

## Abbreviations

AE: Adverse Event; AKI: Acute Kidney Injury; AKIN: Acute Kidney Injury Network; AUC: Area Under the Curve; BNP: B-type Natriuretic Peptide; Cmax: Maximum (peak) concentration; CPAP: Continuous Positive Airway Pressure; CCMDS: Critical Care Minimum Data Set; CVC: Central Venous Catheter; DMEC: Data Monitoring and Ethical Committee; ICU: Intensive Care Unit; MAP: Mean arterial pressure; NGAL: Neutrophil Gelatinase-Associated Lipocalin; PEEP: Positive End-Expiratory Pressure; PerLR: Personal Legal Representative; PIS: Patient Information Sheet; ProLR: Professional Legal Representative; SAE: Serious Adverse Event; ScvVO2: Central Venous Oxygen Saturations; SD: Standard Deviation; SOFA: Serial Organ Failure Assessment; SSC: Surviving Sepsis Campaign; SUSAR: Suspected Unexpected Serious Adverse Reaction; TSC: Trial Steering Committee; VASST: Vasopressin And Septic Shock Trial.

## Competing interests

MS has received fees from Orion Corporation, manufacturer of levosimendan, for sitting on advisory boards and delivering lectures. DM has received fees for sitting on an advisory board for dexmedetomidine, which is manufactured by Orion Corporation. All other authors declare they have no competing interests.

## Authors’ contributions

RMLO conceived the initial trial concept and helped develop the trial design and protocol. GDP, DFM, KDL, AM, MS helped develop the trial design and protocol. AJM and DA carried out the power calculations and helped develop the trial design and protocol. ACG is the chief investigator and takes overall responsibility for all aspects of trial design, the protocol and trial conduct. All authors read and approved the final manuscript.
